# The mediating role of acute respiratory infections in temperature-mortality associations in the Czech Republic, 1982–2019

**DOI:** 10.1007/s00484-025-03119-8

**Published:** 2026-03-04

**Authors:** Ekaterina Borisova, Joan Ballester, Hana Hanzlíková, Eva Plavcová, Jan Kyselý, Jan Kynčl, Aleš Urban

**Affiliations:** 1 Faculty of Environmental Sciences, Czech University of Life Sciences, Kamýcká 129, 165 00 Prague, Suchdol Czech Republic; 2 Institute of Atmospheric Physics of the Czech Academy of Sciences, Prague, Czech Republic; 3 ISGlobal, Barcelona, Spain; 4 National Institute of Public Health, Prague, Czech Republic; 5 Third Faculty of Medicine, Charles University, Prague, Czech Republic

**Keywords:** Temperature-related mortality, Acute respiratory infections, DLNMs, Seasonal mortality, Cold-attributable deaths

## Abstract

Numerous epidemiological studies have shown that non-optimal temperatures and acute respiratory infections (ARIs) contribute to increased mortality. The effect of temperature on mortality is typically estimated using splines of time, which are designed to account for seasonal and longer-term trends, and account for the potential unmeasured confounding, such as the seasonal effect of ARIs. However, the distinct impact of ARIs on mortality, as well as the direct effect of temperature on mortality that is not mediated via ARIs, remains insufficiently understood. This study investigates and quantifies the impact of ARIs on mortality, and the impact of temperature on mortality that is not mediated via ARIs. We used 38-year time-series data from the Czech Republic and applied a distributed lag non-linear model (DLNM) with one and multiple cross-bases to isolate the direct pathways of temperature and ARIs. Our approach allowed us to estimate the fraction of mortality attributable to each factor, providing a clearer understanding of their respective contributions to seasonal mortality patterns. Our findings indicate that ARI activity is a significant mediator of the relationship between temperature and mortality. Since low temperatures increase the risk of both cold-related mortality and ARI incidence, our results indicate that approximately 12% of cold-related deaths during the study period could be attributed to ARI activity. Additionally, an analysis of temporal changes in the combined effect of ARIs and temperature suggested that while the mediating role of ARIs on temperature-related mortality has weakened, the proportion of cold-attributable mortality has remained constant throughout the study period. Our findings are important for understanding both historical trends and future projections of seasonal mortality patterns. They highlight the need for further research on the roles of climatic and individual risk factors in long-term changes in temperature-related mortality, particularly considering the mediating role of ARIs.

**Supplementary Information:**

The online version contains supplementary material available at 10.1007/s00484-025-03119-8.

## Introduction

Observed and projected climate change has increased attention to the relationships between the temperature and human health. Numerous epidemiological studies have investigated the negative impacts of temperature on public health, including mortality and morbidity from cardiovascular, cerebrovascular, and respiratory diseases, and how the intensity of these impacts can vary based on various climatic factors and population characteristics (Achebak et al. 2024; Analitis et al. 2008; Psistaki et al. 2022; Scovronick et al. 2018; Wen et al. 2023). Extreme temperature events, such as cold spells and heatwaves, have been associated with increased mortality rates (Gasparrini et al. 2015; Masselot et al. 2023; Pascal et al. 2018; Romanello et al. 2023), particularly among vulnerable populations (Hajat et al. 2007; Son et al. 2011). Both extreme cold and heat have been consistently linked to the increased risk of cardiovascular, cerebrovascular, and respiratory morbidity and mortality (Chen et al. 2013; Hanzlíková et al. 2015; Hyrkäs-Palmu et al. 2018; Luo et al. 2019; Martínez-Solanas and Basagaña 2019; Stewart et al. 2017; Urban et al. 2014). Furthermore, it is well documented that the impact of heat is immediate and most pronounced among individuals with advanced illnesses, whereas the effect of cold tends to have a longer-lasting effect on mortality (Yu et al. 2012).

Seasonal variations in mortality are not only caused by seasonal variations in environmental conditions, but also by changes in human behavior and the dynamics of infectious diseases (Stewart et al. 2017). While some authors claim that the great majority of excess winter deaths occur due to exposure to ambient cold (Hajat and Gasparrini 2016; von Klot et al. 2012), others argue that increased activity of acute respiratory infections (ARIs) is the key factor responsible for the winter mortality peak (Mølbak et al. 2015; Staddon et al. 2014; Stewart et al. 2017). Some studies suggest that the effects of low temperatures on mortality may be overestimated due to confounding by seasonally related risk factors (such as influenza), especially when long lags are used in analysis (Kinney et al. 2015; Woodward 2014). However, other researchers contend that there is insufficient empirical or theoretical evidence to support these claims (Gasparrini 2016).

The burden of ARIs such as influenza, pneumonia and bronchitis, is another major public concern as these infections spread easily and considerably contribute to global morbidity and mortality (Lytras et al. 2019; Schulger, 2010; WHO 2023). Seasonal influenza is the most prevalent ARI globally. It is primarily caused by two distinct types of influenza viruses, A and B. According to the World Health Organization (WHO), approximately one billion cases of seasonal influenza are registered worldwide annually, with 3–5 million cases resulting in severe illness. Furthermore, it is estimated that influenza is responsible for up to 650 000 respiratory deaths per year globally, with a particularly high impact on crisis-affected populations and among vulnerable demographic groups (Bellos et al. 2010; Mulambya et al. 2020; WHO 2023).

Numerous studies have examined the associations between influenza virus transmission and environmental factors, such as temperature and humidity (Lowen and Steel 2014; Pica et al. 2012). Another group of research has explored the relationship between the seasonal occurrence of infectious diseases and human health, typically without considering concurrent weather effects (Kyncl et al. 2005; Kynčl et al. 2021).

Among studies accounting for the effect of both temperature and ARI activity on human health, most have treated one of the exposures only as a confounder using a simple linear term (Nielsen et al. 2011; Hardelid et al. (2012); von Klot et al. 2012). Other group of research analysed the effect of temperature and ARIs on mortality or morbidity separately (Gasparrini et al. 2015; Paget et al. 2019; Scovronick et al. 2018; Thompson et al. 2003), without assessing their mediating role within a single analytical framework. Moreover, many analyses were limited to short study periods (Murtas and Russo 2019; Nielsen et al. 2019) or focused on specific diagnoses (García-Lledó et al. 2021; Imai et al. 2016; Lee and Yoon 2024; Wen et al. 2023).

However, relatively little is known about the mediating role of ARIs in the effects of temperature on winter mortality when considering the lagged, nonlinear effects of both exposures. Addressing this gap is important for a better understanding of how climatic and infectious factors jointly influence human health and to support the development of effective public health interventions, especially as climate change is expected to increase the frequency and intensity of extreme temperature events (Planton et al. 2008).

In this study we employ a 38-year mortality time series analysis with data from the Czech Republic to investigate the role of ARIs in the temperature-mortality relationship. We aim to quantify the effects of respiratory infections and low temperature on excess mortality and to analyze seasonal and temporal changes in cold- and ARI-related mortality. To account for changes in the actual burden of ARIs and cold on total mortality over time, we employ the distributed lag non-linear (DLNM) model with two cross-basis functions to quantify temporal changes in the fraction and number of deaths attributed to cold and ARIs over 19-year moving periods. The findings of the present study will facilitate a more comprehensive understanding of the role of climate change in the relationships between ARIs, temperature and mortality.

## Methodology

### Data and data sources

Daily counts of all-cause mortality in the Czech Republic covering the 38-year period from 1982 to 2019 were provided by the Czech Statistical Office (CZSO) and the Institute of Health Information and Statistics of the Czech Republic (IHIS CR). As the data collected prior to 1994 were not disaggregated by sex, age or place of residence, we have used nationwide all-cause mortality data for the entire study period. The weekly incidence of ARIs per 100,000 inhabitants for the same period (1982–2019) was obtained from the National Institute of Public Health (NIPH). NIPH defines ARIs as both upper and lower respiratory tract infections, including influenza (Havlickova et al. 2019; Kyncl et al. 2005). With regard to disease classification, the ARI reports used in this study include every clinical diagnosis of acute upper respiratory tract infection (as defined by the International Classification of Diseases, Tenth Revision (ICD-10), codes J00, J02, J04, J05, and J06) and influenza (ICD-10 codes J10.1, J10.8, J11.1, and J11.8). These reports are summary data, in which the individual ICD-10 codes are no longer distinguishable. The study period does not include the COVID-19 pandemic, and COVID-19 incidence and related mortality were intentionally excluded to ensure that estimated long-term relationships between temperature, ARI, and mortality remain unbiased. The original weekly data was decomposed into daily values so that the weekly totals were preserved. The trend within the week takes into account the dynamics of the weekly counts so that the daily values of neighbouring weeks follow on fluently. The daily values obtained in this way were smoothed using locally weighted regression with a 14-day smoothing window (Cleveland 1979). Daily weather data were obtained from the E-OBS dataset, which provides a regular grid of interpolated weather observations from European weather stations at a resolution of 0.25°x0.25° (Cornes et al. 2018). This dataset includes daily mean, maximum and minimum temperatures, daily mean wind speed and relative humidity. Neither dataset had missing values.

### Statistical analysis

#### Analysis of the relationship between temperature, ARIs, and mortality

**Fig. 1:**
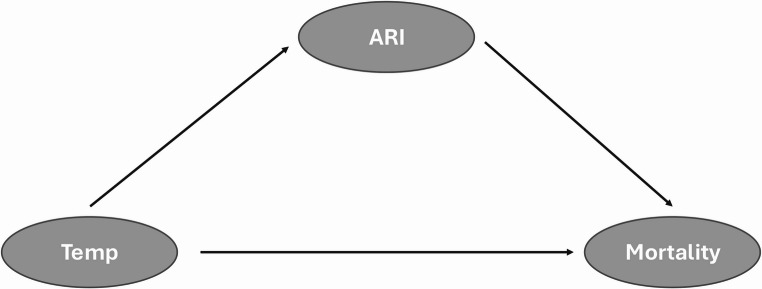
Directed acyclic graph for the ARI-temperature-mortality associations considered in the study

This study proposes that ARIs act as a mediator in the relationship between low temperature and mortality, given the well-established association between temperature and ARIs/influenza incidence, and the fact that many respiratory viruses are more stable and transmissible in cold conditions (Lowen and Steel 2014). The assumed causal pathways between temperature, ARIs, and mortality are depicted in the following directed acyclic graph (Fig. 1). Our aim is to quantify (i) the impact of ARIs on mortality, and (ii) the direct impact of temperature on mortality that is not mediated via ARIs. We note that the effect of temperature on mortality is normally estimated in large-scale epidemiological studies by using splines of time, which are aimed to control for seasonal and longer-term trends, including the unmeasured confounding effect of ARIs.

In the first stage of analysis, in order to estimate the short-term associations between weather variability, mortality, and ARI incidence, we employed a quasi-Poisson regression with Generalized Additive Models (GAM) in distributed lag non-linear models (DLNMs). This framework was developed to capture the non-linear and delayed effects of environmental exposures on health outcomes (Gasparrini et al. 2010). The estimated temperature- and ARIs-mortality associations are further reported as relative risk (RR), which refers to the risk of an outcome associated with a particular exposure compared to a reference level of that exposure.

We used two models to assess the mediating effect of ARI incidence on the links between temperature and mortality. Specifically, M_NoARI represents the traditional approach to model the links between temperature and mortality that does not explicitly incorporate the effect of ARIs. Hence, we hypothesize that this model includes the mediating effect of ARI that is not captured by the spline function for seasonal and long-term changes. The model is described as follows:

$$\:log\left(E\left({Y}_{t}\right)\right)=\alpha\:+cb\left(T\right)+ns\left(Trend,9df\mathrm{*}nyears\right)+{DOW}_{t}$$

where *t* is the day of the observation, $\:{Y}_{t}$ is the daily mortality count observed on the day *t*, $\:E\left(.\right)$ is the expected value, $\:\alpha\:$ is the intercept, $\:cb$ is the cross-basis matrix produced by DLNM, $\:ns\left(Trend\right)$ is a natural spline function with 9 degrees of freedom ($\:df$) multiplied by the number of years ($\:nyears$) to capture long-term patterns related to slow changes in mortality data such as population ageing or improvement of the health care system, and seasonality patterns in mortality. $\:{DOW}_{t}$ is a categorical variable for the day of the week.

The temperature cross-basis was composed of a quadratic B-spline for the exposure-response function, with three internal knots set at the 10th, 75th, and 90th percentiles of the temperature distribution, following previous studies that conducted time-series analyses on similar datasets (Ballester et al. 2024; Gasparrini et al. 2015). For the lag-response function, a natural cubic spline with two internal knots equally distributed in the log-space over lags 0–21 was applied.

M_ARI, on the other hand, incorporates both temperature and ARI incidence effects, and is described as follows:

$$\:log\left(E\left({Y}_{t}\right)\right)=\alpha\:+cb\left(T\right)+cb\left(ARI\right)+ns\left(Trend,9df\mathrm{*}nyears\right)+{DOW}_{t}$$

It replicates the settings of M_NoARI, but additionally includes the ARI cross-basis, thus removing the mediating effect of ARIs from the relationship between temperature and mortality.

The ARI cross-basis was composed of a natural cubic spline with 4 equally spaced knots for the exposure-response function, and a natural cubic spline with a single internal knot for the lag-response function over the same lag period (up to 21 days). The ARI cross-basis settings were chosen based on sensitivity analyses, and the shape of the overall exposure-response curve (Table S1 in Supplementary Material). Most models suggested a linear increase in RR with increased ARI incidence. However, more complex models indicated a larger slope of the RR curve at extreme ARI incidence levels. Since the model with 4 knots indicated a slightly better goodness-of-fit parameter (Generalized Cross Validation score, or GCV) than other models, we considered this setting in the main analysis.

Minimum mortality temperature (MMT) was used as the reference point to estimate the temperature-mortality associations. MMT represents the point at which the overall cumulative exposure-response curve reaches its minimum value, and was determined using the *findmin* function (Tobías et al. 2017). MMTs were estimated for M_NoARI and M_ARI across the entire study period, as well as for each designated subperiod, and were further used as centering values for fitting the exposure-lag-response relationship. For ARI-mortality associations, the zero ARI incidence was used as the reference point. In order to quantify the mediating role of ARIs, temperature effect estimates were derived from M_NoARI and M_ARI, and ARI effect estimates were derived from M_ARI.

Additionally, a sensitivity analysis was conducted to consider the inclusion of other variables to the model. To quantify and compare the models’ fit, we used the GCV score, a measure of goodness of fit that considers the effective degrees of freedom of the model (Wood 2017). The inclusion of several additional variables, such as relative humidity, wind speed and the presence of holidays, was tested in the initial model. However, these variables were excluded from the final analysis, either because they were shown to have non-significant confounding effects (as determined by an ANOVA test) or because they did not significantly improve the model, and we aimed to avoid overfitting (Table S1).

Models described above were firstly applied for the whole study period (1982–2019), and then for the data subsets of 19-year moving subperiods (1982–2000, 1983–2001, …, 2001–2019), to analyze changes in the mediating role of ARI over the study period.

#### Attributable mortality

In the second stage of analysis, we quantified the mortality burden due to cold (i.e. daily mean temperature lower than MMT), heat (i.e. daily mean temperature higher than MMT), and ARIs as the number (AN) and fraction (AF) of total deaths attributable to these factors, using the *attrdl* function in *FluMoDl* package developed by Gasparrini and Leone (2014). AF and AN correspond to the proportion of deaths associated with non-optimal temperature and the incidence of ARIs among all deaths, and their absolute numbers. We adopted a backward calculation of attributable risk measures, linking past (lagged) exposure events to current risks. The attributable mortality was quantified for the whole study period (1982–2019) as well as for the data subsets of 19-year moving periods (see Sect. 1.2.1).

We extended the analysis by calculating monthly AFs due to cold, heat and ARIs in different subperiods, in order to assess temporal changes in the seasonal distribution of temperature- and ARI- attributable mortality.

All the statistical analyses were performed with R software (version 4.3.2) using the packages *dlnm*, *mgcv*, and *FluMoDl*.

## Results

Table 1 presents the summary statistics for weather variables, ARI incidence, and mortality in the Czech Republic from 1982 to 2019. During the study period, there were 4,379,722 all-cause deaths in total, with daily counts ranging from a minimum of 206 to a maximum of 617 deaths. The average daily incidence of ARIs was 143.3 cases per 100,000 inhabitants, peaking at 571 cases. Temperatures fluctuated significantly, with mean daily values ranging from − 20.3 °C to 27.8 °C. The average daily mean temperature was 8.3 °C. Temporal changes in mortality, temperature, and ARI incidence are illustrated in Supplementary Material (Fig. S1).

**Table 1:** Summary statistics of Climatic variables, ARI incidence, and daily deaths in the Czech Republic, during the study period: 1982–2019

Variable	Minimum	P1	P25	Median	Mean	P75	P99	Maximum
Daily Mean temperature (°C)	20.3	-9.8	2.0	8.5	8.3	14.9	23.6	27.8
Daily Maximum temperature (°C)	18.0	-6.3	4.7	12.6	12.3	19.8	30.0	35.2
Daily Minimum temperature (°C)	24.5	-14.4	-0.9	4.3	4.0	9.8	16.6	19.3
Relative humidity (%)	37.9	51.8	71.0	79.4	77.5	85.4	91.4	93.4
Wind (m*s^− 1^)	0.8	1.1	1.7	2.2	2.5	2.9	6.0	10.2
ARI incidence (N per 100,000 inh.)	34.0	47.0	93.0	134.0	143.3	176.0	421.2	571.0
All-cause deaths	206.0	244.0	285.0	309.0	315.6	339.0	434.0	617.0

### Exposure-response associations between temperature, ARIs, and mortality

**Fig. 2:**
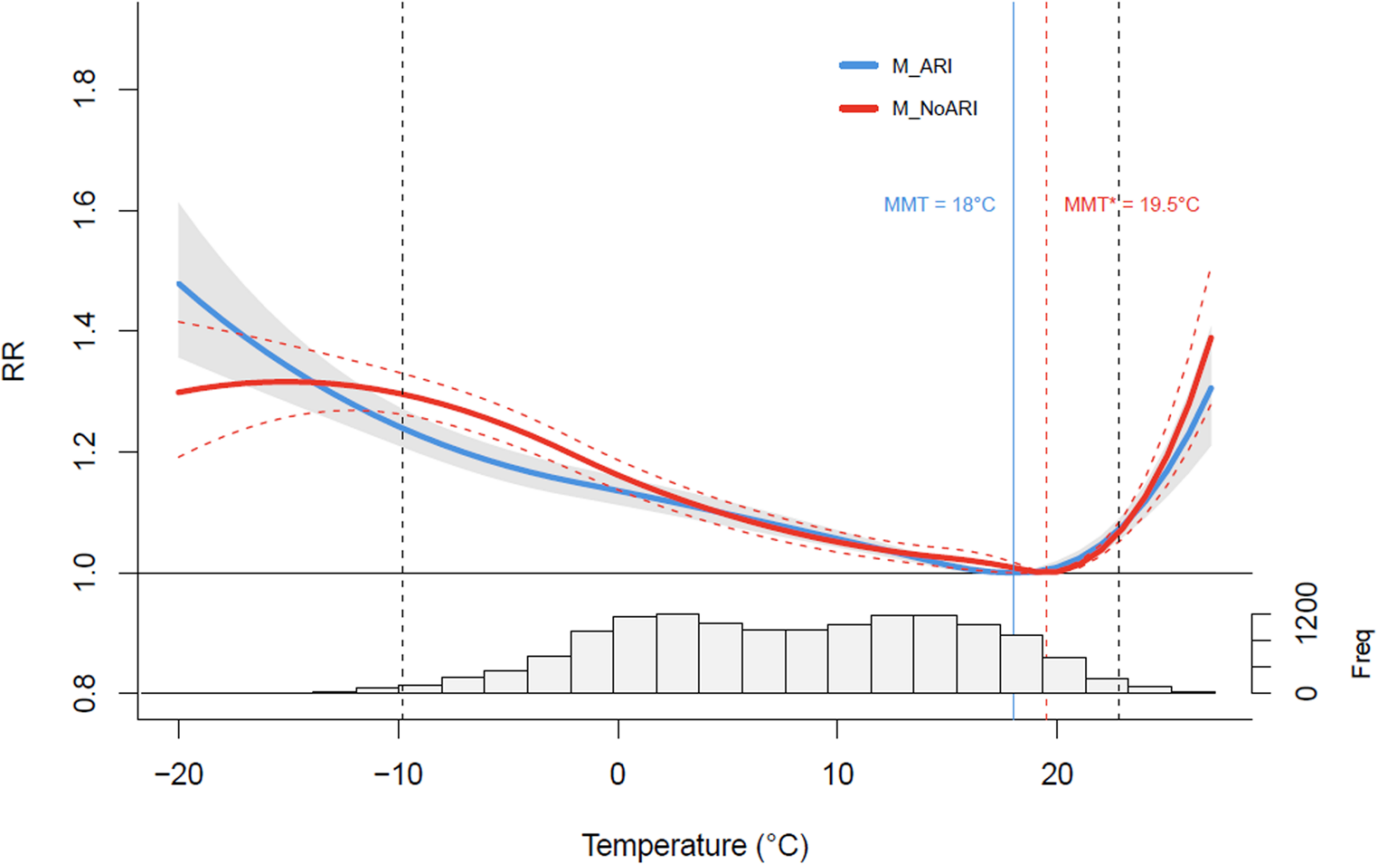
The cumulative associations between mean temperature and all-cause mortality over lag of 0*–*21 days. The red curve is derived from M_NoARI and the blue one is derived from M_ARI. The dashed red and shaded grey curves indicate the 95% CIs.The vertical dashed red and blue lines indicate MMT values (centering points) estimated for both models. The histogram below illustrates the distribution of temperature data, with the 1st and 99th percentiles indicated by black dashed vertical lines

Figure 2 shows the cumulative (over lags of 0–21 days) temperature-mortality curves obtained from the two models. The RR curves show that both low and high temperatures are associated with an increased risk of death compared to MMT, with a typical J shape (a steeper slope for heat than for cold). The estimated MMT was however warmer in M_NoARI compared to M_ARI, with values of 19.5 °C (95% CI: 18.9, 19.8 °C) and 18.0 °C (95% CI: 16.9, 19.2 °C), respectively. The other main difference between the two models was observed at moderate low temperatures, between − 2.1 °C and − 8.2 °C, corresponding to the range where the 95% CI of the RR estimates from the two models do not overlap, and where the RR of mortality was considerably higher in M_NoARI than M_ARI. For example, the RR at −7.0 °C (2.5th percentile of annual temperature distribution) reached 1.27 (95% CI: 1.24, 1.30) for M_NoARI compared to 1.20 (95% CI: 1.17, 1.23) for M_ARI (Table 2).

The RR at the lowest temperatures (between − 13.9 °C and − 20.3 °C) was, on the other hand, lower in M_NoARI than M_ARI. As indicated by the temperature histogram in Fig. 1, only 134 days occurred in this temperature range which resulted in large uncertainty of the RR values at these temperatures.

**Table 2:** Effect of temperature on mortality across percentiles

	Percentile	Temperature (°C)	Overall RR with lag 0–21 days (95% CIs)	
			M_NoARI	M_ARI
1982–2019	1	10	1.30 (1.26, 1.33)	1.24 (1.21, 1.27)
	2.5	7	1.27 (1.24, 1.30)	1.20 (1.17, 1.23)
	5	5	1.24 (1.21, 1.27)	1.18 (1.15, 1.20)
	97.5	22	1.04 (1.03, 1.05)	1.05 (1.03, 1.06)
	99	24	1.13 (1.10, 1.16)	1.12 (1.09, 1.15)
1982–2000	1	10	1.32 (1.27, 1.37)	1.22 (1.18, 1.26)
	2.5	8	1.30 (1.26, 1.34)	1.19 (1.15, 1.23)
	5	5	1.26 (1.22, 1.30)	1.15 (1.12, 1.19)
	97.5	21	1.02 (1.01, 1.03)	1.04 (1.02, 1.07)
	99	23	1.10 (1.06, 1.13)	1.10 (1.07, 1.14)
2001–2019	1	9	1.27 (1.22, 1.32)	1.25 (1.20, 1.30)
	2.5	7	1.25 (1.21, 1.29)	1.23 (1.18, 1.27)
	5	4	1.22 (1.18, 1.26)	1.19 (1.15, 1.24)
	97.5	22	1.03 (1.02, 1.04)	1.03 (1.02, 1.05)
	99	24	1.11 (1.08, 1.14)	1.11 (1.07, 1.14)

**Fig. 3:**
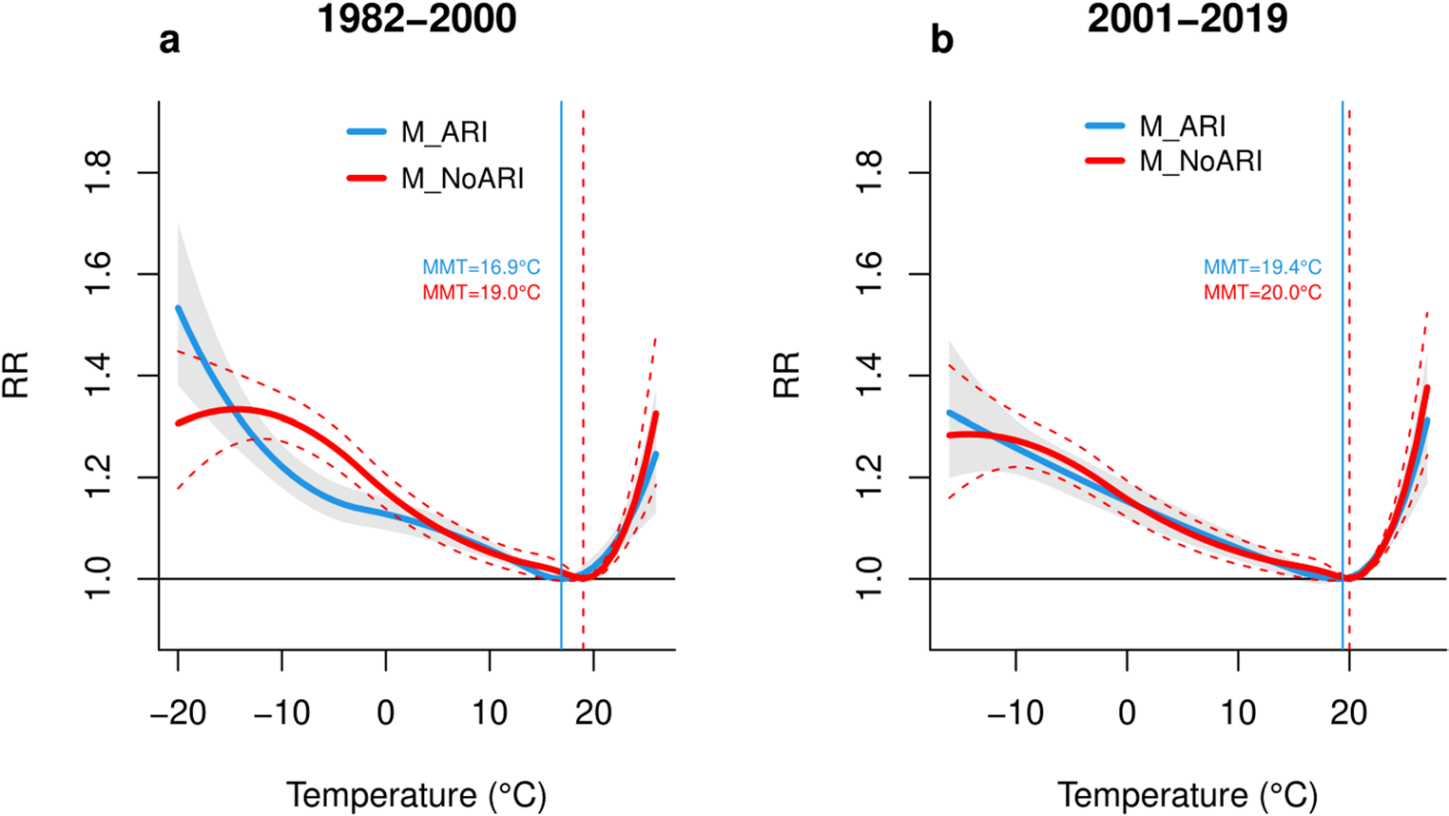
Comparison of cumulative temperature-mortality curves for 1982*–*2000 (**a**) and 2001*–*2019 (**b**) subperiods

Figure 3 presents a comparison of the exposure-response curves in the first (1982–2000) and last (2001–2019) 19-year subperiods. Table 2 shows the RR values at selected percentiles. The results reveal a decrease in RRs for cold and, on the other hand, a slight increase in RR for heat during the last subperiod (see Table 2 for specific values). Furthermore, the gap between the exposure-response curves derived from the M_NoARI and M_ARI diminished towards the more recent years, suggesting a weakening effect of ARIs on mortality.

**Fig. 4:**
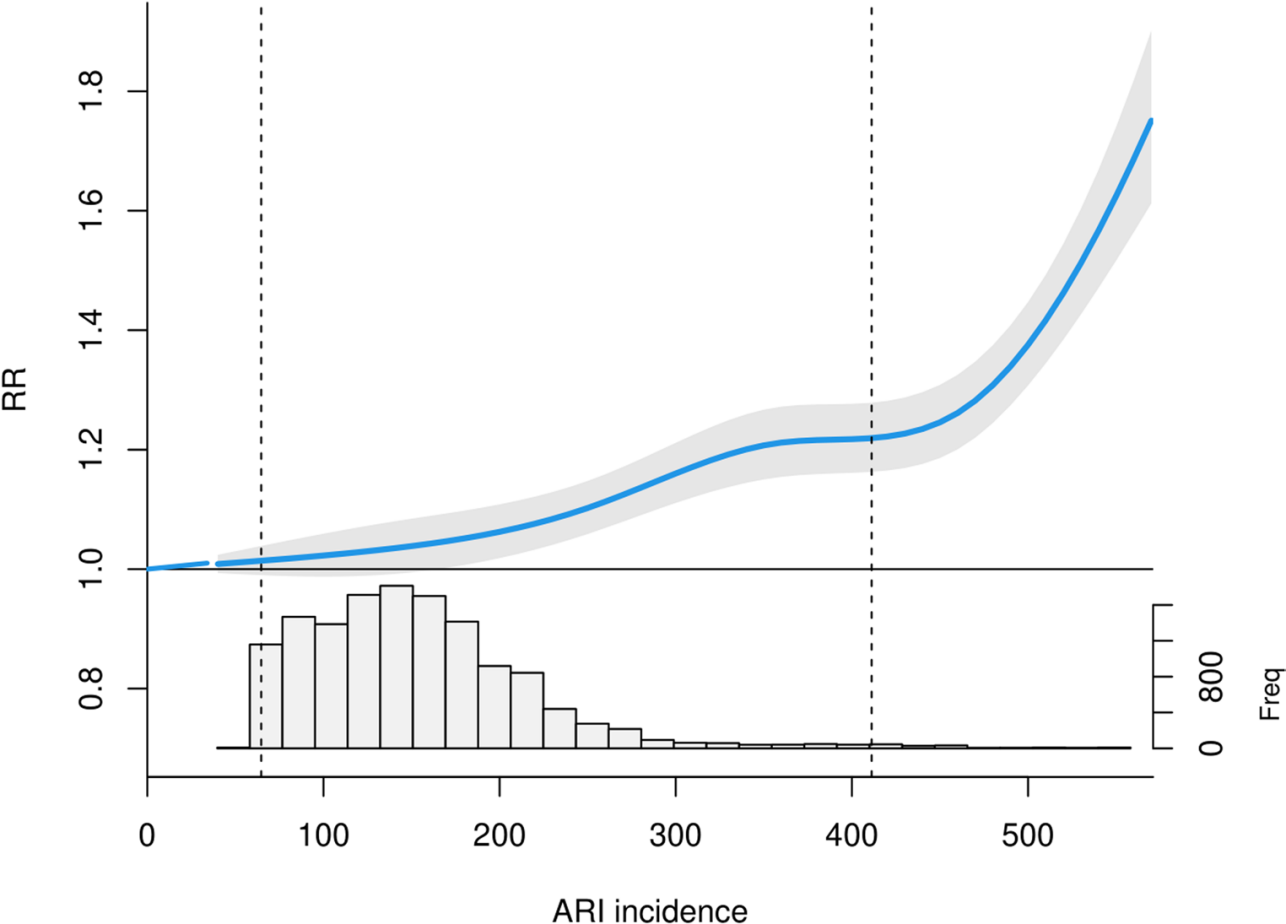
The cumulative association between ARI incidence and all-cause mortality over lag of 0*–*21 days (M_ARI). The histogram below illustrates the distribution of ARI incidence, with the 1st and 99th percentiles indicated by black dashed vertical lines

Indeed, the weakening impact of ARIs on mortality was also documented in the exposure-response curves for ARI. The overall cumulative ARI-mortality relationship is shown in Fig. 4 and detailed in Table 3. The graph demonstrates that increased incidence of ARI is associated with a higher RR. At lower ARI incidences (approximately up to 180 cases per 100,000 population, equivalent to the 76.8th percentile), the RR is 1.05 (95%CI: 1.01, 1.10), suggesting no major increase in the risk. The RR becomes particularly positive at the ARI incidence of 250 cases (equivalent to the 93.7th percentile), with a RR of 1.01 (95%CI: 1.06, 1.15). The most substantial increase is observed above 505 (99.8th percentile) cases, with the RR at 510 cases equal to 1.42 (95% CI: 1.34, 1.49). The ARI effect estimates for different percentiles are represented in Table S2.

**Table 3:** ARI effect estimates for different ARI incidence levels

ARI incidence:	Overall RR 0–21 days (95% CI)		
	1982–2019	1982–2000	2001–2019
50	1.01 (0.99, 1.03)	1.03 (1.00, 1.05)	1.00 (0.97, 1.04)
200	1.06 (1.02, 1.11)	1.11 (1.05, 1.17)	1.02 (0.96, 1.09)
300	1.16 (1.11, 1.21)	1.22 (1.15, 1.29)	1.11 (1.03, 1.20)
400	1.22 (1.16, 1.28)	1.30 (1.22 1.38)	1.10 (1.01, 1.19)
500	1.38 (1.31, 1.45)	1.46 (1.37, 1.56)	
550	1.62 (1.52, 1.74)	1.68 (1.55, 1.82)	

**Fig. 5:**
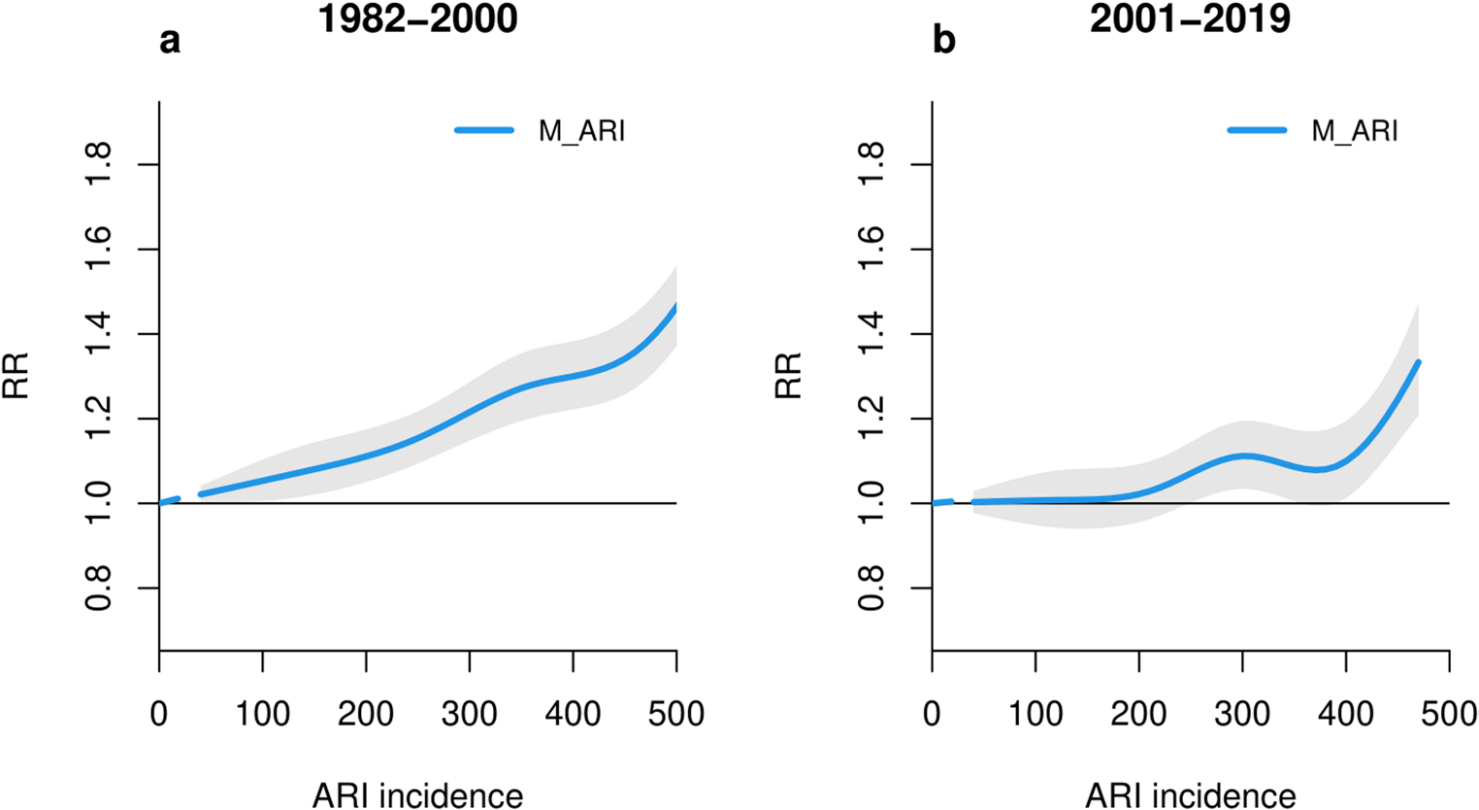
Comparison of cumulative ARI-mortality curves for 1982*–*2000 (**a**) and 2001*–*2019 (**b**) subperiods

Figure 5 illustrates the temporal evolution of the association between ARIs and mortality, with data spanning the periods 1982–2000 (Fig. 5a) and 2001–2019 (Fig. 5b). A decrease in the RR attributable to ARIs was observed in the latter subperiod. To illustrate, the RR at the 95th percentile of ARI incidence was 1.20 (95% CI: 1.14, 1.27) for the initial subperiod (with an incidence of 290 cases), and 1.07 (95% CI: 1.00, 1.14) for the subsequent subperiod (with an incidence of 250 cases).

### Attributable mortality risk

Table 4 illustrates the attributable fraction (%) and number (deaths) due to non-optimum temperatures and ARI incidence across the entire study period, analysed using the M_NoARI and M_ARI models. The mortality burden resulting from exposure to cold and ARIs was found to be greater than that associated with heat. In line with the results for RRs, incorporating the ARI incidence into the M_ARI had a considerable impact on the estimation of temperature-related mortality, particularly with regard to cold-related deaths. Accordingly, while 7.82% (95% eCI: 6.50, 9.03%) of the total annual deaths were attributable to cold by M_NoARI, the estimated mortality attributable to cold was 6.90% (95% eCI: 5.87, 7.90%) according to M_ARI. In terms of total attributable deaths, these results suggest that approximately 12% (40,206) of deaths attributable to cold according to M_NoARI could be attributed to the mediating effect of ARIs.

Furthermore, as the burden of mortality attributable to ARIs was 4.35% (95% eCI: 0.85, 7.71%) according to M_ARI, an additional 150,108 deaths could be attributed to the combined effect of cold and ARI in M_ARI compared to the M_NoARI model.

Surprisingly, temperature-attributable mortality estimated by M_ARI was also larger in case of heat: 0.36% (eCI: 0.26, 0.45%) compared to 0.31% (95% eCI: 0.25, 0.38%) for M_NoARI, which is an equivalent to 1950 heat-attributable death difference. However, the difference was relatively small and not significant according to the largely overlapping eCIs.

**Table 4:** Mortality attributable to cold, heat, and ARI in the observational period: 1982–2019 (95% empirical CI)

	M_NoARI		M_ARI		
	Cold	Heat	ARI	Cold	Heat
AF (%)	7.82 (6.50, 9.03)	0.31 (0.25, 0.38)	4.35 (0.85, 7.71)	6.90 (5.87, 7.90)	0.36 (0.26, 0.45)
AN	342 561 (286 388, 396 625)	13 744 (11 145, 16 566)	190 314 (34 886, 341 113)	302 355 (250 452, 349 250)	15 694 (11 133, 19 496)

**Fig. 6:**
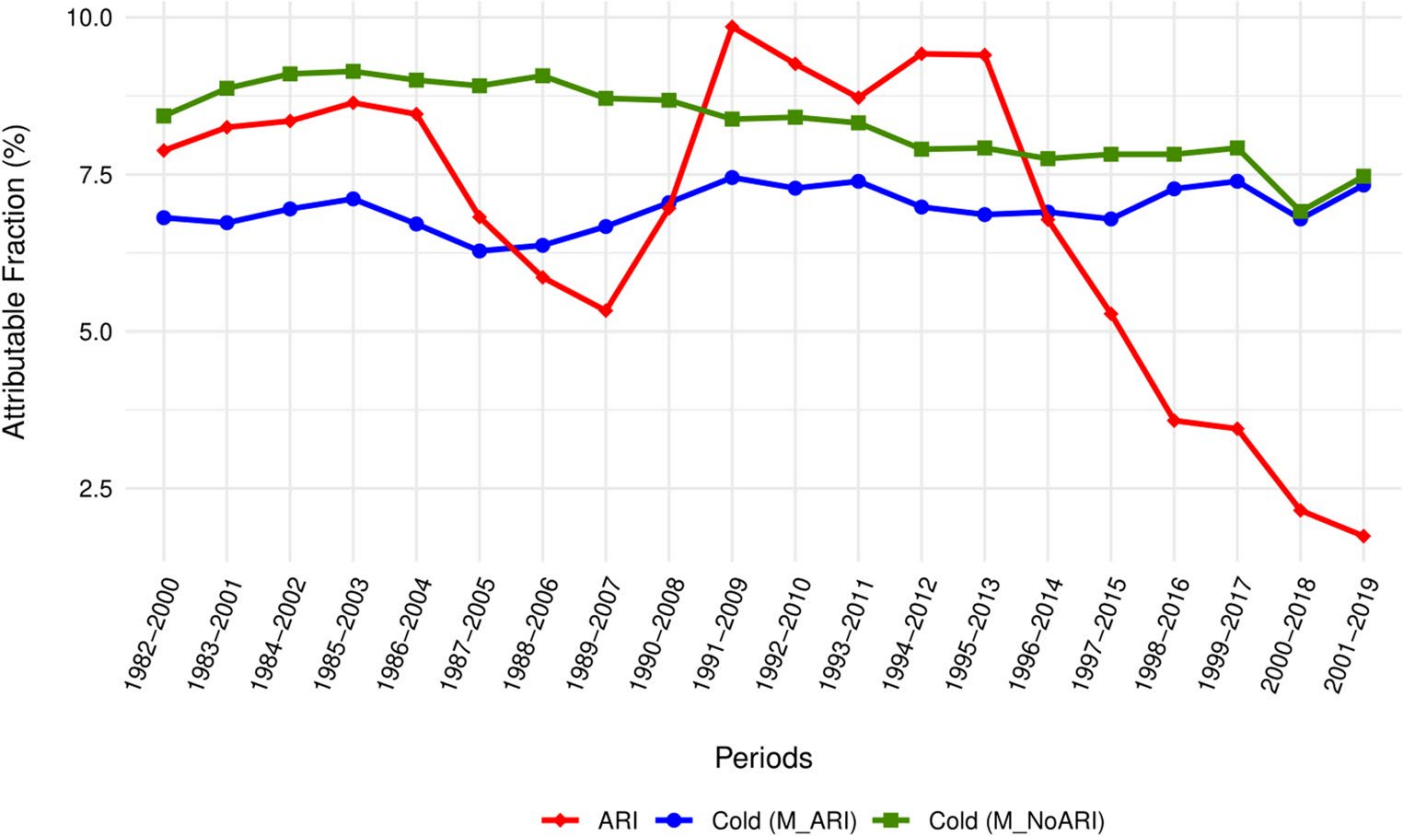
Time evolution of AF of total deaths due to AR and cold estimated from M_NoARI and M_ARI

Figure 6 illustrates temporal changes in AF due to cold and ARIs for the moving 19-year subperiods from 1982 to 2019. The results show that the fraction of deaths due to cold from M_NoARI has consistently decreased over time from 8.43% (95% eCI: 6.50, 10.16%) in 1982–2000 to 7.47% (95% eCI: 5.60, 9.32%) in 2001–2019.

On the other hand, the fraction of deaths due to cold from M_ARI was consistently lower than that derived from M_NoARI and remained relatively constant over time. It was 6.81% (95% eCI: 5.33, 8.43%) in 1982–2000, and 7.33% (95% eCI: 5.43, 9.13%) in 2001–2019.

The mortality burden attributable to ARIs has undergone a noticeable decline over time. In 1982–2000, ARIs accounted for 7.88% (95% eCI: 3.23, 12.41%) of all-cause mortality. However, this fraction decreases significantly over time, reaching 1.74% (95% eCI: -4.35, 7.83%) in 2001–2019. A particularly notable decline in the AF of total deaths due to ARIs is observed starting from the period 1997–2015, once the winter season of 1995/1996, characterized by the highest influenza-related mortality, is excluded from the analysis (Fig. S3).

Temporal changes in the number of all-cause mortality attributable to exposure to cold and ARIss are detailed in Table S3 and illustrated in Fig. S2.

**Fig. 7:**
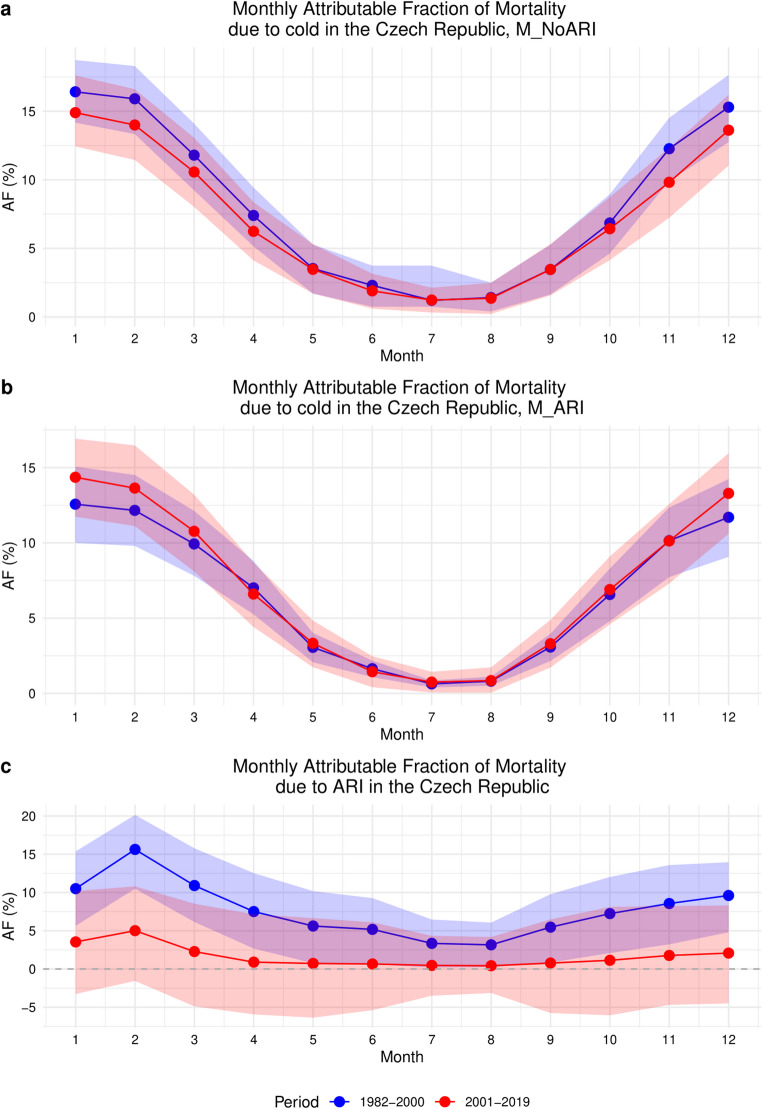
Monthly AFs due to cold and ARIs derived from M_NoARI and M_ARI. AFs are estimated with 95% empirical CI

Figure 7 depicts the monthly AF of mortality due to cold (i.e. daily mean temperature lower than annual MMT; Fig. 7a and b) and ARI (Fig. 7c) derived for the first and last 19-year subperiods.

As expected, both models (M_NoARI and M_ARI) show a consistent seasonal pattern of cold-related AF with maximum values in the winter months (in the following order: January, February, December) in both subperiods (Table S4). In line with the previous results, the M_NoARI model revealed a decreasing trend in cold-attributable mortality (AF in January reaching 16.42% (95% eCI: 14.17, 18.74%) in the first subperiod vs. 14.90% (95% eCI: 12.44, 17.62%) in the last subperiod. In contrast, the M_ARI model demonstrated a slight increase in winter mortality attributable to cold. The AF in January rose from 12.57% (95% eCI: 10.00, 15.07%) during the period 1982–2000 to 14.36% (95% eCI: 11.74, 16.92%) in 2001–2019.

Additionally, the AF of mortality specifically attributable to ARIs shows a considerable decrease between the two subperiods, particularly during the winter season, when ARI epidemics are most prevalent. February was the peak month in both subperiods regarding the ARI-related AF. However, while in 1982–2000 the February AF peaks at approximately 15.63% (95% eCI: 10.49, 20.14%) it dropped to 5.01% (95% eCI: -1.56, 10.80%) in 2001–2019.

## Discussion

### Overall mediating effect of ARIs

The objective of this study was to examine the impact of ARIs on mortality, as well as the impact of temperature on mortality that is not mediated via ARIs. While several studies have explored the relationship between ARIs, temperature and mortality (Hardelid et al. 2012; Nielsen et al. 2011, 2019), this study advances the previous research by applying the state-of-art methodology in epidemiological modelling – DLNMs with one and two cross-basis functions to evaluate the ARIs as a potential mediator in the temperature-mortality association, including the lagged effects of both exposure variables. By examining ARIs as a key intermediary, our study offers a more nuanced understanding of the impact of temperature fluctuations on mortality, particularly in the winter season. These insights can inform the development of more effective public health strategies for mitigating the health risks associated with climate variability.

Our findings align with existing research demonstrating an association between low temperatures, influenza activity and higher mortality rates. Already, Imai et al. (2016) found that ischemic heart disease (IHD) deaths from direct cold exposure peaked within 10 days, while those related to influenza infection peaked at 4–6 days and continued for up to 30 days, suggesting that influenza infection is a plausible factor contributing to the delayed link between cold exposure and cardiovascular mortality. Our results extend that understanding by quantifying the mediating role of ARIs in the relationship between temperatures and mortality, particularly during the cold season. Indeed, our study revealed that, during the study period (1982–2019), the overall number of cold-related deaths in the Czech Republic was approximately 40,000 lower in a model adjusted for the effect of ARIs (M_ARI), compared to the basic model (M_NoARI) that considered only the effect of temperature. These results suggest that a considerable part (12%) of the mortality risk attributable to low temperatures in M_NoARI was captured by the inclusion of the ARI cross-basis in M_ARI and thus can be attributed to the mediating effect of ARIs. This finding highlights the importance of considering the role of ARIs in temperature-mortality studies.

Another key observation is the time-dependent nature of ARI-mediated mortality. Over recent years, the mortality risk associated with ARIs has become less pronounced, likely due to factors such as warmer winters, the emergence of viruses causing less severe disease and associated lower mortality, and improved health care.

### Warmer winters

It is well documented that low temperatures can increase the prevalence of ARIs. Cold and dry weather has been shown to impair immune responses and facilitate the transmission of respiratory viruses, which can lead to higher morbidity and mortality rates (D’Amato et al. 2018; Lee and Yoon 2024; Yu et al. 2024). However, in recent years, we have observed a notable shift in weather patterns in Central Europe with milder winters becoming prevalent and decreased winter temperature variability (Copernicus Climate Change Servise 2023; Lorenz et al. 2019; Pokorná et al. 2018). This warming trend has potentially contributed to a reduction in the incidence of ARIs (Mäkinen et al. 2009; Ballester et al. 2016). This shift, associated with warming winters, suggests that over time, as winters become less severe, the impact of ARIs on mortality burden and its mediating role in the temperature-related mortality may have diminished.

Another key observation is that MMT is constantly higher in the M_NoARI model because it does not account for the mediating effects of ARIs, which amplify cold-related mortality. By including an ARI cross-basis, the M_ARI model separates the effects of ARIs, reducing the apparent mortality at colder temperatures and shifting the MMT downward. This highlights the significant role ARIs play in shaping the temperature-mortality relationship.

### Changing patterns in dominant influenza viruses

Additionally, the mortality rates attributable to ARIs are highly dependent on the specific types or subtypes of circulating respiratory viruses during a given period. The impact of different influenza virus strains on health outcomes can vary considerably. For example, as reported by Lytras et al. (2019), the A/H3N2 influenza subtype is associated with significantly higher mortality rates compared to the A/H1N1 and influenza B viruses. In our time-series analysis (Fig. S3 in the supplementary material), we observe a shift in the circulation of influenza subtypes after 2000, with A/H1N1 becoming more frequent. At the same time, A/H3N2 continues to correlate with more severe mortality spikes during its dominant seasons. This shift in the prevalence of subtypes may contribute to the overall reduction in ARI-related mortality in recent years, as A/H1N1 generally causes milder outcomes compared to the more virulent A/H3N2 subtype. This factor contributed to the particularly pronounced decline in ARI-related mortality, after excluding the winter season of 1995/1996, which was characterized by exceptionally severe A/H3N2 epidemics and exceptionally high excess mortality rates (Kwok et al. 2017; Kyncl et al. 2005).

Our findings indicate that the changing patterns in dominant influenza viruses may have also considerably affected the overall trends in cold-related mortality trends observed in our study. A comparison of the decreasing trend in cold-attributable mortality burden in M_NoARI model, with no distinct decline in the M_ARI model suggests that cold-related mortality remained stable over the study period when the model was adjusted for the mediating effect of ARIs. However, the observed decrease in the effect of ARIs is crucial, as it contributes to the observed decline in cold-related mortality burden estimated from the M_NoARI model.

### Improved healthcare

The diminished impact of ARIs on mortality in recent years can also be explained by the socioeconomic progress and improved life expectancy in the Czech Republic. Following the Velvet Revolution in 1989 and the fall of communism, significant changes in lifestyle, healthcare, and public health systems contributed to better health outcomes across the population. Reforms in healthcare access, improvements in nutrition, and higher standards of living have collectively increased resilience to diseases like ARIs and reduced the impact of environmental factors, such as cold, on mortality rates. All these factors led to a significant rise in the life expectancy in the Czech Republic between 1990 and 2010s (Song et al. 2018; Bryndová et al. 2023; The Global Economy 2023).

Our findings have important implications for both historical observations and future projections of seasonal patterns in mortality and encourage further investigation of the role of climatic and socioeconomic factors in long-term changes in links between temperature-related mortality, taking into account the mediating role of ARIs.

### Limitations

Our results suggest that the decreasing trends in mortality fraction attributable to cold observed between 1982 and 2019 have been driven by the weakening effect of ARIss in the later subperiods (Fig. 6). This might be related to the climatic and socioeconomic factors mentioned above. However, one important limitation must be acknowledged when interpreting the final results. In 2009, the Government of the Czech Republic introduced a new health care policy that considerably changed the sickness benefits (Ministry of Labour and Social Affairs, 2008). Under the new policy, no sick pay is available for the first three working days of sick leave (European Social Policy Network 2019). This change likely influenced illness reporting, particularly for mild to moderate ARIs, as some people unable to afford unpaid sick leave might choose not to report their illness. Consequently, the actual incidence of ARIs has been very likely underestimated in official data, especially for less severe cases not requiring immediate medical attention.

Although the sensitivity analysis did not reveal a significant effect of the new health care policy in the statistical model (Table S1), this underreporting may have resulted in an inaccurate representation of the ARI incidence data employed in the study, which could subsequently affect the modeling results regarding the mediating role of ARIs in the temperature-mortality relationship. Despite this limitation, the ARI incidence data represent the most consistent and longest time series of ARI activity in the Czech Republic. Therefore, it would be beneficial for future research to address policy-related factors and consider ways to adjust for potential underreporting to obtain a more accurate assessment of ARI incidence and its impact on mortality.

Another limitation of this study is the lack of consideration of influenza vaccination rates. Although many studies show the positive effect of the influenza vaccination rate on declining influenza-related morbidity and mortality (Song et al. 2018), historical data indicate that vaccination coverage in the Czech Republic has consistently fallen below the WHO, European, and national targets of 75% for older adults and individuals with chronic illnesses (Havlickova et al. 2019). Since recent estimates suggest that vaccination coverage among these high-risk groups is approximately 25%, with no notable improvement in recent years (WHO 2023), we assume that this factor had a negligible impact on our results.

Additionally, while this study uses nationwide data, limiting the ability to capture regional variations, it still provides highly robust modeling outcomes, offering a comprehensive overview at the national level.

## Conclusions

This study analyzed a 38-year mortality time series (1982–2019) from the Czech Republic to investigate the mediating role of ARIs in temperature-related mortality. By examining the interaction between ARI epidemics and low temperatures, we provided new insight into how respiratory infections such as influenza affect the links between ambient temperature and mortality, particularly in the cold season. We further quantified the effects of respiratory infections and low temperatures on excess mortality and analyzed seasonal and temporal changes in cold- and ARI-related mortality.

Our findings indicate that ARIs play a significant mediating role in the relationship between temperature and mortality. Given that low temperatures increase the risk of both cold-related mortality and ARI incidence, our results suggest that a significant proportion (approximately 12%) of the cold-related mortality during the study period was attributable to ARI activity. Additionally, an analysis of temporal changes in the compound effect of ARIs and temperature suggests that as the mediating effect of ARIs on temperature-related mortality has weakened, the cold-attributable mortality fraction has remained constant throughout the study period. These findings have important implications for both the historical observations and future projections of seasonal patterns in mortality and encourage further investigation of the role of climatic and socioeconomic factors in long-term changes in links between temperature-related mortality, taking into account the mediating role of ARIs.

## Supplementary Information

Below is the link to the electronic supplementary material.

Supplementary Material 1

## Data Availability

The mortality data underlying this article were provided by the Institute of Health Information and Statistics of the Czech Republic (https://www.uzis.cz/index-en.php) by permission. ARI incidence data were provided by the National Institute of Public Health by permission. Weekly ARI incidence data are publicly available at (https://szu.gov.cz/publikace-szu/data/akutni-respiracni-infekce-chripka/). The R code for replicating the statistical analysis is available at Zenodo: 10.5281/zenodo.16994743. Input health data used in the code will be shared on request to the corresponding author with permission of the Institute of Health Information and Statistics of the Czech Republic and the National Institute of Public Health. The temperature data underlying this study were derived from an E-OBS data set, available at https://cds.climate.copernicus.eu/datasets/insitu-gridded-observations-europe?tab=overview.
