# Supplementary material for: The mediating role of acute respiratory infections in temperature-mortality associations in the Czech Republic, 1982–2019
Source: Int J Biometeorol. 2026 Mar 4;70(3):84. doi: 10.1007/s00484-025-03119-8 (PMC12960470; doi:10.1007/s00484-025-03119-8)
Supplement: Supplementary file 1 — Supplementary Material 1 [file 484_2025_3119_MOESM1_ESM.docx]

Supporting Information for

**The Mediating Role of Acute Respiratory Infections in Temperature-Mortality Associations in the Czech Republic, 1982 – 2019**

**Ekaterina Borisova^1,2^, Joan Ballester^3^, Hana Hanzlíková^2,4^, Eva Plavcová^2^, Jan Kyselý^1,2^, Jan Kynčl^5,6^, Aleš Urban^1,2^**

^1^Faculty of Environmental Sciences, Czech University of Life Sciences, Prague, Czech Republic,

^2^Institute of Atmospheric Physics of the Czech Academy of Sciences, Prague, Czech Republic,
^3^ISGlobal, Barcelona, Spain,
^4^Institute of Geophysics of the Czech Academy of Sciences, Prague, Czech Republic,
^5^National Institute of Public Health, Prague, Czech Republic,
^6^Third Faculty of Medicine, Charles University, Prague, Czech Republic

Corresponding authors:

Ekaterina Borisova [borisova@fzp.czu.cz](mailto:borisova@fzp.czu.cz)

**Contents of this file**

**Table S1.** Sensitivity analysis comparing two models, M_NoARI (excluding ARI data) and M_ARI (including ARI data), based on several key modeling metrics: R-squared (R-sq), Generalized Cross-Validation (GCV), and the percentage of deviance explained.

**Table S2.** ARIs effect estimates for different percentiles computed from M_ARI.

**Table S3.** Number of all-cause mortality attributable to cold and ARI computed from M_NoARI and M_ARI.

**Table S4.** Monthly AF due to cold and ARI computed from M_NoARI and M_ARI.

**Figure S1.** Temporal trends in temperature, ARI incidence, and mortality in the Czech Republic in 1982-2019.

**Figure S2.** Temporal changes in number of all-cause mortality attributable to cold and ARI computed from M_NoARI and M_ARI.

**Figure S3.** Temporal trends in mortality and ARI epidemics by dominant virus type, highlighting the frequent dominance of the A/H3N2 virus and its association with higher mortality spikes.

| **Table S1**. Sensitivity analysis comparing two models, M_NoARI (excluding ARI data) and M_ARI (including ARI data), based on several key modeling metrics: R-squared (R-sq), Generalized Cross-Validation (GCV), and the percentage of deviance explained. | | | | | | |
| --- | --- | --- | --- | --- | --- | --- |
| Modelling choices | M_NoARI | | | M_ARI | | |
|  | R-sq | GCV | Deviance explained (%) | R-sq | GCV | Deviance explained (%) |
| Main model | 0.772 | 1.2718 | 77.6 | 0.787 | 1.2038 | 78.8 |
| Knots for exposure-response (Temp): 10th, 50th, and 90th | 0.77 | 1.2824 | 77.4 | 0.787 | 1.2094 | 78.8 |
| Knots for exposure-response (Temp): 3 equally spaced knots  Knots for lag-response (Temp):  2 equally spaced knots | 0.77 | 1.2843 | 77.4 | 0.787 | 1.2092 | 78.8 |
| Knots for exposure-response (Temp): 2 equally spaced knots.  Knots for lag-response (Temp):  2 equally spaced knots | 0.77 | 1.2843 | 77.4 | 0.786 | 1.211 | 78.7 |
| Lag period: 14 days | 0.77 | 1.2843 | 77.4 | 0.7 | 1.2096 | 78.8 |
| Cubic spline for exposure-response and lag-response (ARI) | 0.771 | 1.2739 | 77.6 | 0.787 | 1.2066 | 78.8 |
| Linear function for exposure-response and lag-response (ARI) | 0.771 | 1.2739 | 77.6 | 0.785 | 1.2131 | 78.6 |
| Natural spline with 1df for exposure-response (ARI) | 0.771 | 1.2739 | 77.6 | 0.785 | 1.2121 | 78.7 |
| Df/year for seasonal control: 7 | 0.763 | 1.3116 | 76.6 | 0.782 | 1.2251 | 78.2 |
| Df/year for seasonal control: 8 | 0.768 | 1.2871 | 77.2 | 0.785 | 1.2103 | 78.6 |
| Control for changes in the health care policy | 0.772 | 1.2718 | 77.6 | 0.787 | 1.2038 | 78.8 |
| Control for humidity and wind | 0.773 | 1.266 | 77.7 | 0.788 | 1.2 | 78.9 |

| **Table S2.** ARIs effect estimates for different percentiles (M_ARI) | | | |
| --- | --- | --- | --- |
|  | Percentile | Incidence of ARIs | Overall RR with lag 0-21 days (95% CIs) |
| 1982–2019 | 1 | 47 | 1.01 (0.99, 1.03) |
|  | 5 | 55 | 1.01 (0.99, 1.03) |
|  | 50 | 134 | 1.03 (0.99, 1.08) |
|  | 95 | 264 | 1.12 (1.07, 1.16) |
|  | 99 | 421 | 1.22 (1.17, 1.28) |
| 1982–2000 | 1 | 47 | 1.02 (1.00, 1.05) |
|  | 5 | 56 | 1.03 (1.00, 1.06) |
|  | 50 | 133 | 1.07 (1.01, 1.13) |
|  | 95 | 286 | 1.2 (1.13, 1.27) |
|  | 99 | 445 | 1.33 (1.25, 1.42) |
| 2001–2019 | 1 | 47 | 1.00 (0.97, 1.03) |
|  | 5 | 53 | 1.00 (0.97, 1.04) |
|  | 50 | 135 | 1.01 (0.97, 1.08) |
|  | 95 | 254 | 1.08 (1.01, 1.15) |
|  | 99 | 360 | 1.08 (1.00, 1.17) |

| **Table S3.** Number of all-cause mortality attributable to cold and ARI computed from M_NoARI and M_ARI (95% eCI) | | | | |
| --- | --- | --- | --- | --- |
| Periods | ARI | Cold (M_NoARI) | Cold (M_ARI) | Total deaths |
| 1982–2000 | 183 153 (74 560, 278 341) | 195 974 (153 375, 240 540) | 158 337 (122 654, 192 269) | 2 323 521 |
| 1983–2001 | 189 858 (89 076, 285 231) | 203 991 (161 528, 241 453) | 154 831 (120 306, 189 166) | 2 300 511 |
| 1984–2002 | 189 863 (77 370, 288 258) | 206 892 (169 121, 245 398) | 158 069 (122 003, 193 146) | 2 274 280 |
| 1985-2003 | 194 783 (88 169, 289 447) | 205 880 (165 414, 245 188) | 160 233 (125 678, 191 204) | 2 253 380 |
| 1986–2004 | 188 515 (74 045, 282 511) | 200 528 (158 524, 242 631) | 149 516 (114 662, 184 601) | 2 228 821 |
| 1987–2005 | 152 041 (45 038, 252 838) | 196 295 ( 153 082, 237 072) | 138 490 (103 838, 171 809) | 2 204 171 |
| 1988–2006 | 127 737 ( 20 516, 224 578) | 197 784 (153 196, 236 873) | 139 934 (102 353, 174 140) | 2 181 368 |
| 1989–2007 | 115 192 (11 780, 215 565) | 188 250 (144 740, 228 098) | 144 150 (109 235, 175 302) | 2 160 310 |
| 1990–2008 | 148 763 ( 40 500, 249 646) | 185 452 (145 098, 225 495) | 150 666 (116 885, 183 705) | 2 137 511 |
| 1991–2009 | 208 436 (103 901, 310 426) | 177 382 (138 112, 216 134) | 157 544 (125 969, 188 416) | 2 115 766 |
| 1992–2010 | 194 319 (76 876, 301 655) | 176 552 (134 965, 215 073) | 152 795 (120 921, 184 298) | 2 098 320 |
| 1993–2011 | 181 846 (63 963, 295 862) | 173 366 (131 193, 215 511) | 153 989 (120 119, 186 160) | 2 084 831 |
| 1994–2012 | 195 525 (75 079, 319 956) | 163 949 (123 296, 202 280) | 144 760 (111 854, 177 971) | 2 074 835 |
| 1995–2013 | 194 188 (61 918, 305 522) | 163 720 (122 161, 201 627) | 141 704 (107 939, 173 125) | 2 066 623 |
| 1996–2014 | 139 272 (15 008, 256 258) | 159 197 (126 077, 188 222) | 141 673 (107 930, 175 639) | 2 054 375 |
| 1997–2015 | 108 347 (-10 890, 234 133) | 160 475 (125 622, 194 210) | 139 391 (106 168, 172 213) | 2 052 766 |
| 1998–2016 | 73 304 (-47 837, 201 645) | 160 126 (126 544, 193 429) | 148 815 (114 073, 181 614) | 2 047 772 |
| 1999–2017 | 70 672 (-55 338, 198 389) | 162 237 (127 357, 197 411) | 151 371 (118 068, 186 706) | 2 049 688 |
| 2000–2018 | 44 166 (-80 934, 166 145) | 141 855 (99 587, 178 315) | 139 340 ( 101 567, 171 929) | 2 052 840 |
| 2001–2019 | 35 699 (-82 923, 153 972) | 153 567 (116 539, 193 554) | 150 773 (110 729, 187 258) | 2 056 201 |

| **Table S4**. Monthly AF due to cold and ARI computed from M_NoARI and M_ARI (95% empirical CI) | | | | | | |
| --- | --- | --- | --- | --- | --- | --- |
| Month | Cold (M_NoARI) | | Cold (M_ARI) | | ARI (M_ARI) | |
|  | 1982–2000 | 2001–2019 | 1982–2000 | 2001–2019 | 1982–2000 | 2001–2019 |
| January | 16.42 (14.17, 18.74) | 14.9 (12.44, 17.62) | 12.57 (10.0, 15.07) | 14.36 (11.74, 16.92) | 10.5 (5.66, 15.38) | 3.54 (-3.26, 10.19) |
| February | 15.91 (13.34, 18.3) | 14.0 (11.45, 16.61) | 12.16 (9.8, 14.51) | 13.64 (11.12, 16.48) | 15.63 (10.49, 20.14) | 5.01 (-1.56, 10.80) |
| March | 11.8 (9.23, 14.1) | 10.57 (8.03, 13.07) | 9.93 (7.81, 12.13) | 10.77 (8.1, 13.21) | 10.91 (6.15, 15.78) | 2.28 (-4.88, 8.53) |
| April | 7.4 (5.15, 9.44) | 6.24 (4.12, 8.37) | 7.01 (5.26, 8.80) | 6.60 (4.41, 8.69) | 7. 51 (2.67, 12.53) | 0.9 (-5.93, 7.16) |
| May | 3.53 (1.7, 5.29) | 3.47 (1.71, 5.31) | 3.06 (2.07, 4.02) | 3.33 (1.74, 4.84) | 5.62 (0.76, 10.18) | 0.73 (-6.36, 6.65) |
| June | 2.3 (0.74, 3.74) | 1.9 (0.6, 3.15) | 1.64 (1.08, 2.18) | 1.44 (0.41, 2.47) | 5.18 (0.62, 9.27) | 0.66 (-5.38, 6.11) |
| July | 1.21 (0.74, 3.74) | 1.23 (0.23, 2.13) | 0.63 (0.39, 0.86) | 0.75 (0.06, 1.44) | 3.35 (0.21, 6.47) | 0.47 (-3.49, 4.34) |
| August | 1.41 (0.4, 2.5) | 1.36 (0.22, 2.49) | 0.81 (0.51, 1.11) | 0.85 (0.05, 1.73) | 3.16 (0.24, 6.07) | 0.43 (-3.13, 4.19) |
| September | 3.46 (1.64, 5.27) | 3.46 (1.57, 5.31) | 3.08 (2.15, 3.96) | 3.3 (1.72, 4.88) | 5.47 (0.85, 9.78) | 0.78 (-5.75, 6.48) |
| October | 6.85 (4.63, 8.95) | 6.43 (4.15, 8.74) | 6.57 (4.8, 8.29) | 6.9 (4.59, 9.1) | 7.24 (2.08, 12.03) | 1.14 (-6.05, 8.12) |
| November | 12.27 (9.9, 14.52) | 9.82 (7.23, 12.33) | 10.15 (7.73, 12.35) | 10.13 (7.3, 12.58) | 8.56 (3.22, 13.58) | 1.77 (-4.69, 8.23) |
| December | 15.3 (12.75, 17.65) | 13.62 (11.04, 16.2) | 11.70 (9.07, 14.24) | 13.29 (10.6, 15.96) | 9.61 (4.8, 13.96) | 2.08 (-4.49, 8.31) |


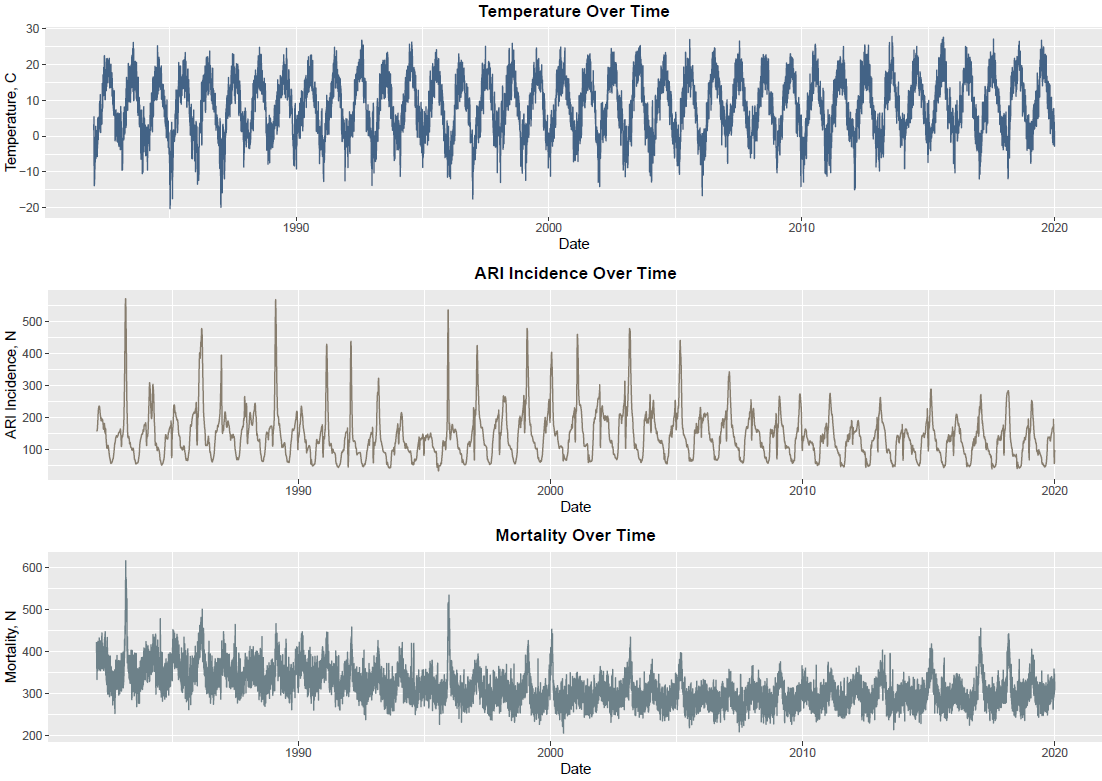


**Fig. S1.** Temporal trends in temperature, ARI incidence, and mortality in the Czech Republic in 1982-2019


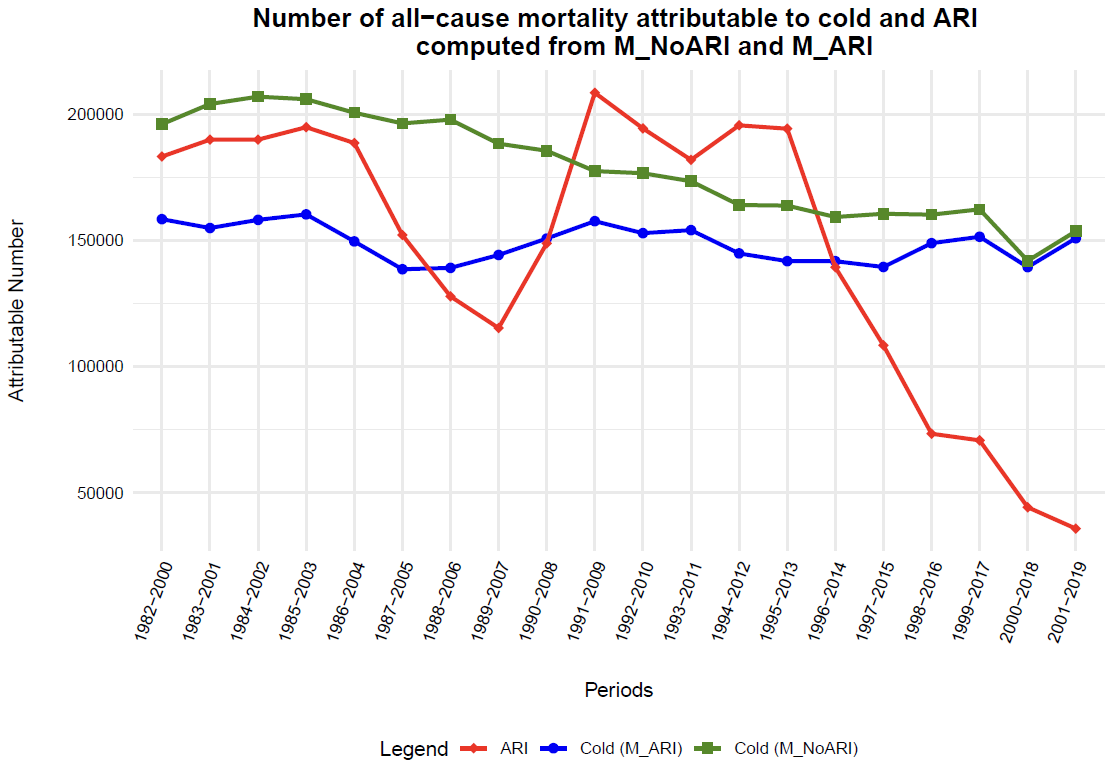


**Fig. S2.** Number of all-cause mortality attributable to cold and ARI computed from M_NoARI and M_ARI.


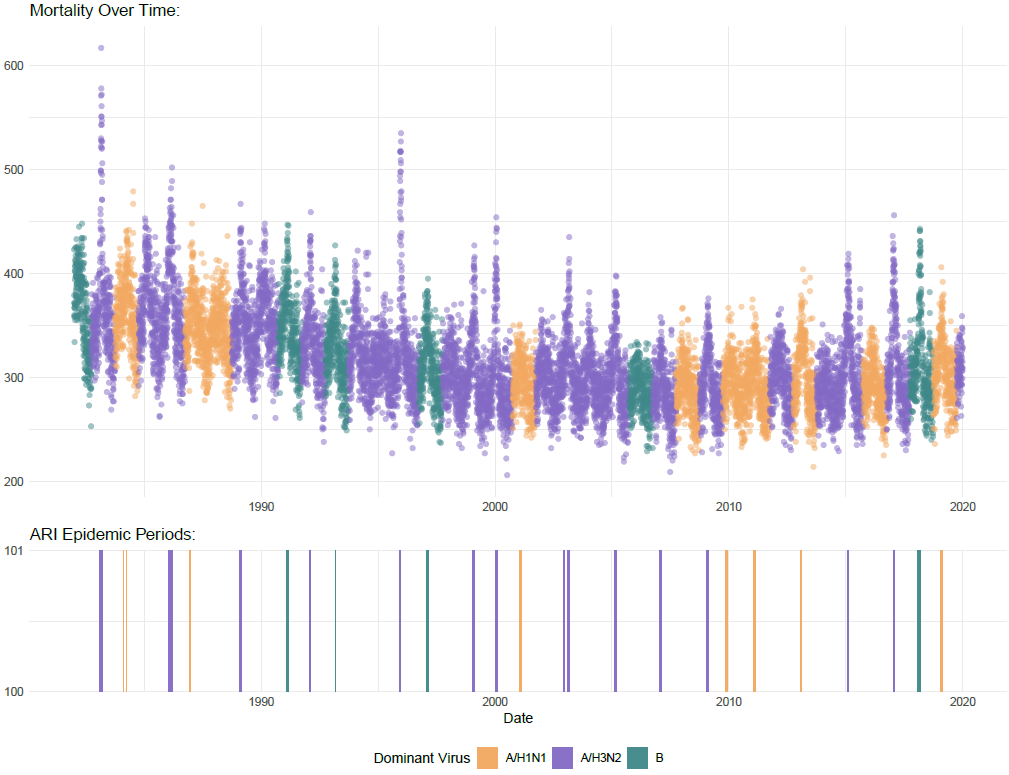


**Fig. S3.** Temporal trends in mortality and ARI epidemics by dominant virus type, highlighting the frequent dominance of the A/H3N2 virus and its association with higher mortality spikes.
